# Cut-off values of coronary risk factors in women with hypothyroidism

**DOI:** 10.6026/973206300200762

**Published:** 2024-07-31

**Authors:** Deepika Srivastava, Latheef S.A.A., Venkatramana P.

**Affiliations:** 1 Discipline of Anthropology, School of Social Sciences, Indira Gandhi National Open University, New Delhi, India; 2 Department of Genetics, Osmania University, Hyderabad, India

**Keywords:** coronary risk, hypothyroidism

## Abstract

Women with hypothyroidism are at higher risk of cardiovascular diseases and the consequent mortality. It is not known whether cut-off values of coronary risk factors in women with hypothyroidism are the same as healthy women. This may help to initiate interventions as early to prevent cardiovascular mortality. Therefore, this study was conducted to determine the cut-off values of coronary risk factors in women with hypothyroidism. One hundred women patients with hypothyroidism were compared with 100 healthy controls. Significantly higher mean body mass index (BMI), waist circumference (WC), triglycerides and low-density lipoprotein cholesterol (LDL-C) were observed in women with hypothyroidism than without it. All variables showed an area under curve (AUC) value of >0.6 in receiver operator characteristic curve (ROC) analysis, and similar to healthy women.

## Background:

Globally, 70 people per 1000 individuals suffer from abnormal thyroid function [1]. Thyroid disorders are the common endocrine abnormalities in India and among them 42 million suffer from thyroid diseases [2]. Among thyroid disorders, hypothyroidism was found to be affecting one in ten individuals in India and its prevalence was found to be two-fold higher than western populations. Deficiency of the thyroid hormones (thyroxine (T4) and triiodothyronine (T3)) was observed in patients with hypothyroidism [3]. About 1/3 of patients with hypothyroidism in all age groups were found to be undetected and untreated in India [4]. The prevalence of hypothyroidism is 11% in India, and it was found to be higher in inland than in coastal states, in females than males and older than all age groups [2, 5, 6]. Because hypothyroidism exacerbates other risk factors such as hypertension and hyperlipidemia, it raises the risk of cardiovascular morbidity and mortality [7, 8-9]. Therefore, it is of interest to report whether the cut-off values for predicting the risk of coronary risk factors in women with hypothyroidism are the same as healthy women or not.

## Methods:

One hundred women patients with hypothyroidism (TSH levels of >5.5µIU/ml) were compared with healthy women (<5.5 µIU/ml). The age of both patients and healthy controls ranged from 20 to 60 years. Patients were drawn from the Asian Institute of Medical Sciences, Faridabad and Holy Family hospital, Delhi, whereas, health controls were recruited from the clinics located in Faridabad, who came for health check-up. Height was measured with an anthropometer and weight with a calibrated weighing machine. BMI was calculated using the formula weight /(height in m2). WC at the region between the iliac crest and coastal margins was measured using a non-elastic tape. The fasting blood sample was drawn and assayed with an auto analyser for glucose, total cholesterol (total-C), triglycerides, HDL cholesterol (HDL-C) and LDL-C. Systolic (SBP) and diastolic blood pressure (DBP) were measured using a calibrated digital blood pressure monitor.

## Statistical analyses:

Collected data were entered into the Excel spreadsheet. Data were analyzed with SPSS software and a trial version of Medcalc software.

## Results:

Mean values of quantitative variables were compared between patients with hypothyroidism and healthy controls and presented in Table 1. Significantly higher mean BMI, WC, triglycerides and LDL-C were observed in women with hypothyroidism than without it. Predictive ability and cut-off values of variables contributing to CRFs were evaluated using ROC analyses and presented in Table 2. In all variables, AUC was>0.6 and significant, suggesting that these variables with obtained cut-off values are able to predict the risk of coronary risk factors.

## Discussion:

In the present study, we defined coronary risk factors (hypertension (SBP>140/DBP >90 mmHg), obesity (BMI>25 Kg/m2), high total-C (>200mg/dl), high triglycerides (>150mg/dl), high fasting glucose (>126 mg/dl), high LDL-C (>130mg/dl), high waist circumference (>80 cm) and low HDL-C (<50mg/dl)) in women with hypothyroidism using continuous variables (total-C, glucose, triglycerides, BMI, LDL-C, WC, HDL-C, SBP and DBP) of the present study. All continuous variables were found to predict the risk of coronary risk factors with AUC of >0.6 as shown by ROC results of Table 2. The obtained cut-off values except triglycerides were slightly lower with respective units (BMI: 0.1, WC:0.2, SBP:1, DBP:1, total-C:2, LDL-C:0.4, HDL-C:0.02 and glucose:1) than cut-off values used for defining the coronary risk factors suggesting that the cut-off values for predicting the risk of CRFs are same even in patients with hypothyroidism. In present study, significantly higher prevalence of general (73% vs 52%,p=0.002), central obesity or high WC (91% vs 76%,p=0.006) high LDL-C(44% vs 25%,p=0.005) and high triglyceride (38% vs 25%,p=0.048) (data not shown) was observed in patients with hypothyroidism when compared to healthy controls. No evidence is available in the literature for comparison of findings of the present study. Thyroid hormone receptors are found in myocardium and endothelium and regulate the homeostasis of the cardiovascular system. Myocardial dysfunction (depression of left ventricular systolic and diastolic dysfunction at rest and exercise) and accelerated atherosclerosis formation (abnormal lipid metabolism, changes in blood pressure, endothelial dysfunction and insulin resistance), are the two proposed mechanisms contributing to the risk of cardiovascular disease in patients with hypothyroidism. Higher prevalence of diabetes, hypertension and lipid abnormalities was reported in patients with hypothyroidism [10]. The present study showed higher prevalence of general and central obesity, high LDL-C (p<0.01) and triglyceride (p<0.05) levels in patients with hypothyroidism when compared to the healthy controls.

## Conclusion:

A higher prevalence of obesity and lipid abnormalities in patients with hypothyroidism than in healthy controls suggests clustering of coronary risk factors. Initiation of early interventions against lipid abnormalities and reducing weight in patients with hypothyroidism can reduce the morbidity and mortality from cardiovascular diseases.

## Figures and Tables

**Table 1:** Descriptive statistics of patients with hypothyroidism and healthy controls among adult non-Pregnant women

**Variables**	**Hypothyroidism Patients(n=100) Mean ± Standard deviation**	**Healthy controls (n=100) Mean ± Standard deviation**	**p value**
Age (Years)	41.61 ± 9.15	42.13 ± 10.62	0.628
Body mass index (Kg/m^2^)	26.91 ± 3.54	25.21 ± 3.12	0.001
Waist circumference(cm)	92.99 ± 10.37	86.39 ± 8.91	0
Systolic blood Pressure (mmHg)	124.35 ± 15.47	122.24 ± 11.51	0.132
Diastolic blood Pressure (mmHg)	81.74 ± 9.21	81.08 ± 6.94	0.418
Glucose (mg/dl)	103.68 ± 41.37	102.18 ± 35.60	0.899
Total cholesterol (mg/dl)	178.05 ± 37.91	174.72 ± 37.35	0.514
Triglycerides (mg/dl)	138.49 ± 49.82	131.98 ± 75.06	0.02
High density lipoprotein cholesterol (mg/dl)	44.79 ± 6.66	46.33 ± 7.33	0.217
Low density lipoprotein cholesterol (mg/dl)	130.24 ± 35.38	114.13 ± 31.42	0.001
cm: centimetre; Kg: Kilogram; m^2^: meter squared; mg: milligrams; dl: decilitre

**Table 2:** Cut-off values, area under curve, confidence interval, sensitivity, specificity, positive and negative predictive values of continuous variables

**Variable**	**Cut-off value**	**AUC**	**95% CI, p value**	**Sen**	**Spe**	**PPV**	**NPV**
BMI(Kg/m^2^)	>24.9	0.981	0.951-0.995 <0.0001	97.6	98.67	98.2	98.2
WC (cm)	>79.8	1	0.982-1.000 <0.0001	100	100	100	100
SBP (mmHg)	>139	0.986	0.958-0.997 <0.0001	100	94.15	92.6	100
DBP(mmHg)	>89	0.993	0.969-1.000 <0.0001	100	93.09	91.3	100
Total-C (mg/dl)	>198	1	0.982-1.000 <0.0001	100	100	100	100
Triglycerides (mg/dl)	>150	1	0.982-1.000 <0.0001	100	100	100	100
LDL-C (mg/dl)	>129.6	1	0.982-1.000 <0.0001	100	100	100	100
HDL-C (mg/dl)	≤49.98	1	0.982-1.000 <0.0001	99.34	100	100	99.8
Glucose (mg/dl)	>125	1	0.981-1.000 <0.0001	100	99.43	99.2	100
BMI: Body mass index; Kg: Kilogram; m^2^: Meter squared; WC: Waist circumference; cm: centimetre; SBP: Systolic blood pressure;
DBP: Diastolic blood pressure; mmHg: millimetre mercury; C: Cholesterol; LDL: Low density lipoprotein; HDL: High density lipoprotein;
Sen: sensitivity; Spe: specificity; PPV: positive predictive value; NPV: negative predictive value; AUC: area under curve;
CI: confidence interval.
